# Examining Regulatory Classifications and Social Determinants of Health in Telehealth-Mediated ADHD Prescription Fulfillment Barriers

**DOI:** 10.3390/medsci14060651

**Published:** 2026-10-09

**Authors:** Yunshu Yang, Weijian Qin, Wenyu Zhang, Taiwo Opeyemi Aremu

**Affiliations:** 1 Division of Health Policy and Management, University of Minnesota, Minneapolis, MN 55455, USA; 2 Department of Population Health Science, Weill Cornell Medicine, New York, NY 10065, USA; weq4001@alumni.weill.cornell.edu; 3 Whiting School of Engineering, Johns Hopkins University, Baltimore, MD 21218, USA; wzhang@jhu.edu; 4 Department of Clinical and Social Pharmaceutical Sciences, College of Pharmacy, Touro University California, 1310 Club Dr., Vallejo, CA 94592, USA; taremu@touro.edu; 5 Division of Environmental Health Sciences, School of Public Health, University of Minnesota, 420 Delaware Street SE, Minneapolis, MN 55455, USA

**Keywords:** telehealth, attention-deficit/hyperactivity disorder (ADHD), prescription fulfillment, stimulant shortage, social determinants of health (SDOH), health equity

## Abstract

**Background:** Telehealth has expanded remote access to ADHD care, yet a prescription issued remotely must still be filled at a pharmacy—and whether telehealth resolves these downstream fulfillment barriers remains unclear. Federal regulatory distinctions between Schedule II controlled stimulants and non-controlled ADHD pharmacotherapies create structurally divergent procurement experiences, particularly under ongoing nationwide stimulant shortages. This study examined whether prescription fulfillment difficulty differs by medication regulatory classification among adults using telehealth for ADHD care, and identified social determinants of health (SDOH) associated with access difficulty. **Methods:** Data were drawn from Round 2 of the NCHS Rapid Surveys System (RSS-2; October–November 2023). The analytic sample comprised 141 adults with a current ADHD diagnosis who reported pharmacotherapy use and telehealth utilization for ADHD care, classified as controlled-exposed (*n* = 95) or non-controlled-exposed (*n* = 46) by medication regulatory class. Chi-square tests, multivariable logistic regression, and five machine learning algorithms were applied across 19 SDOH variables. **Results:** Prescription access difficulty was substantially more prevalent among controlled-exposed than non-controlled-exposed participants (75.8% vs. 45.7%, *p* < 0.001). Multivariable logistic regression identified controlled medication exposure (coefficient = 2.155, *p* < 0.001), difficulty paying medical bills (coefficient = 1.409, *p* = 0.034), and income (coefficient = 0.145, *p* = 0.042) as significant correlates. Random Forest permutation importance ranked controlled medication exposure and having a usual place for care as the two strongest predictors, although these exploratory rankings warrant caution given the small held-out test set (*n* = 28). Notably, all 13 participants reporting non-prescribed online medication purchases also reported access difficulty through formal channels, a pattern consistent with displacement toward unregulated supply chains. **Conclusions:** Prescription fulfillment barriers were strongly stratified by medication regulatory classification, with financial strain further compounding access difficulty. As federal telemedicine flexibilities approach their 31 December 2026, expiration, regulatory deliberations should weigh downstream fulfillment constraints—including the Schedule II refill prohibition under shortage conditions—alongside upstream telehealth access.

## 1. Introduction

Adult attention-deficit/hyperactivity disorder (ADHD) is increasingly recognized as a persistent neurodevelopmental condition with a global prevalence of 6.8% rather than solely a childhood disorder [1,2]. When not adequately managed, ADHD can impair executive functioning, educational attainment, occupational performance, and psychosocial wellbeing while imposing substantial economic burdens on patients and healthcare systems [3,4]. Although pharmacotherapy remains a central component of clinical management, the benefit of treatment depends on the patient’s ability to obtain medications reliably after a prescription is issued [5].

The United States ADHD pharmacological market is heavily stratified by the Controlled Substances Act (CSA), which dictates prescribing rigidity, monitoring protocols, and supply stability across three distinct regulatory tiers. Specifically, Schedule II stimulants, including widely prescribed methylphenidate and amphetamine formulations, are subject to the most stringent oversight due to a high potential for abuse and dependence [6,7]; these agents strictly prohibit refills, necessitating a new prescription for every individual issuance [8]. Governed by rigid annual DEA production quotas, this primary stimulant supply chain has faced severe nationwide shortages since 2022, with manufacturers utilizing only approximately 70% of allotted production quotas and generating an estimated market deficit of roughly 1 billion doses in 2022 alone [9].

In contrast, Schedule IV agents—such as modafinil and armodafinil—operate under a lower documented abuse potential [6,10], allowing up to five refills within a six-month window [11] and remaining largely insulated from the severe shortages characterizing the primary stimulant market. Entirely outside this framework, non-controlled pharmacotherapies like atomoxetine, viloxazine, clonidine, guanfacine, bupropion, and venlafaxine carry negligible abuse risks and are immune to DEA quotas or stringent remote-prescribing mandates.

This structural bifurcation between controlled and non-controlled substances creates divergent patient experiences. Controlled substances require in-person evaluations under the Ryan Haight Act [12], rendering remote prescribing entirely contingent upon temporary federal flexibilities extended through 31 December 2026 [13], and prescribing them requires clinicians to maintain active DEA registrations in the patient’s specific jurisdiction [6], posing cross-state administrative barriers; non-controlled alternatives are immune to both constraints.

The controlled drug regulatory landscape further exhibits acute policy sensitivity, where federal quota or enforcement adjustments instantly propagate across both Schedule II and IV categories [14], whereas non-controlled alternatives are entirely shielded from these policy-driven fluctuations. Consequently, this study categorizes Schedule II and IV substances into a single controlled-exposed cohort to evaluate their shared structural vulnerability to federal regulatory shifts compared to non-controlled ADHD treatments. This combined classification reflects the exposure construct under study—shared federal controlled-substance status and its common remote-prescribing constraints, including DEA registration requirements and the Ryan Haight Act’s in-person evaluation mandate—rather than uniformity in refill rules or shortage exposure, which, as noted above, differ between the two schedules. In addition, too few participants used Schedule IV agents alone to support a separate analytic stratum; the controlled-exposed group therefore predominantly reflects Schedule II stimulant users. The survey field period (October–November 2023) captures a uniquely policy-dense juncture: pandemic-era flexibilities had temporarily suspended the Ryan Haight Act’s in-person mandates [12], permanent rulemaking remained under active federal deliberation [15], and stimulant dispensing had surged by 45.5% between 2012 and 2021—including a greater than 10% spike during pandemic telehealth expansions—against a supply chain already in deficit [9].

On an individual level, these structural frictions potentially intersect with baseline socioeconomic and logistical headwinds defined within social determinants of health (SDOH) frameworks [16]. Documented evidence indicates that transportation barriers impact healthcare access for 3% to 67% of sampled populations [17], with population-level data from the National Health Interview Survey confirming that 5.8 million Americans (1.8%) annually delay medical care due to transportation deficits—a barrier showing higher prevalence among Hispanic individuals, low-income populations, and Medicaid recipients [18]. Such logistical and economic constraints frequently correlate with diminished clinical persistence: approximately 50% of patients fail to take chronic pharmacotherapies as prescribed due to multi-level barriers [19], and meta-analytic evidence establishes that adverse social determinants significantly reduce the odds of medication adherence, with notable non-adherence patterns linked to food insecurity and housing instability [20].

Although this emerging evidence describes adult ADHD clinical care, less is known about how social and regulatory factors jointly shape patient-reported difficulties obtaining medications. National RSS-2 estimates indicated that nearly one-half of adults with current ADHD utilized telehealth for ADHD-related services, and 71.5% of those taking stimulants reported fulfillment difficulties due to unavailability [5]. Similarly, claims-based studies have tracked telemedicine-based stimulant initiation and prescribing volumes across the pandemic era, revealing that while telehealth flexibilities successfully sustained treatment initiation, these billing data were structurally limited to clinicians’ electronic prescribing records [21]. A recent machine-learning analysis further identified financial hardship, age, and race as key SDOH correlates of telehealth utilization itself among adults with current ADHD [22]. Consequently, this literature has focused primarily on upstream telehealth utilization and aggregate prescribing volumes, leaving the downstream logistical frictions of physical medication fulfillment under asymmetric statutory frameworks under-examined.

To address these gaps, our study provides a policy-relevant quantitative analysis of downstream ADHD prescription fulfillment difficulties among contemporary telehealth recipients. Rather than modeling complex causal mechanisms or estimating formal regulatory effects, this exploratory study addresses a straightforward research question: Among adults with current ADHD who utilized telehealth for their care, are physical prescription fulfillment difficulties more prevalent among those using controlled medications than among those using non-controlled therapies, and which multi-level social determinants of health factors characterize patients at the highest risk of these delivery constraints? Methodologically, standard multivariable logistic regression is deployed as an interpretable descriptive benchmark model, complemented by machine learning classifiers to support exploratory feature importance mapping within highly correlated, small-sample, and high-dimensional SDOH domains. These fulfillment barriers fall squarely within the operational purview of managed care organizations and pharmacy benefit managers, which shape medication access through formulary design, network pharmacy coverage, and utilization management; the findings accordingly speak to ongoing federal deliberations as telemedicine flexibilities approach their 31 December 2026 expiration [13].

This paper is organized as follows: Section 2 describes the data source, study population, medication exposure classification, outcome, and analytic methods. Section 3 reports the descriptive, regression, and machine-learning findings. Section 4 interprets these findings against the statutory asymmetry between controlled and non-controlled ADHD pharmacotherapies and delineates the study’s limitations. Section 5 concludes.

## 2. Methods

This study utilized de-identified, publicly available data from Round 2 of the National Center for Health Statistics (NCHS) Rapid Surveys System (RSS-2) to investigate how SDOH shape difficulty in obtaining ADHD prescriptions among adults currently diagnosed with ADHD, with particular attention to whether such difficulty differs between users of controlled and non-controlled medications. Machine learning (ML) approaches were applied to identify key predictors and capture complex patterns among covariates. Because the analysis poses minimal risk to participants and relied entirely on de-identified records, Institutional Review Board (IRB) approval was not required. Reporting followed the STROBE guideline for cross-sectional studies [23].

### 2.1. Data Source

Data were drawn from Round 2 of the NCHS Rapid Surveys System (RSS-2), administered in October to November 2023. The survey targeted a nationally representative sample of U.S. adults aged 18 years and older through two probability-based online panels, NORC’s AmeriSpeak and Ipsos’s KnowledgePanel, using identical instruments. Reported cumulative panel response rates were 3.8% and 4.0%, respectively, and the overall RSS-2 completion rate was 37.2% [24]. The majority of respondents completed the survey via computer-assisted web interviews (CAWI), with a smaller share completing computer-assisted telephone interviews (CATI). Of the 7046 adults who participated, a final analytic cohort of 141 individuals who met the eligibility criteria was retained for this study. Additional details on RSS and the contributing panels are available on the CDC website [25].

### 2.2. Measures

#### 2.2.1. Study Population

The study population was restricted to adults who reported a current diagnosis of ADHD and exposure to ADHD pharmacotherapy, and who had ever received telehealth services for ADHD. This targeted restriction serves as a deliberate strategy to evaluate the operational boundaries of current telemedicine policy flexibilities. By holding the care delivery modality constant across the sample, this design isolates downstream physical procurement barriers from upstream clinical access variations, allowing evaluation of how statutory drug classifications interact with individual SDOH under relaxed federal telemedicine regulations. Participants were classified into two mutually exclusive groups based on the regulatory class of the medication used: (1) the controlled-exposed group, comprising respondents who reported any use of Schedule II stimulants or Schedule IV agents (Armodafinil, Modafinil); and (2) the non-controlled-exposed group, comprising respondents who reported using only non-controlled medications, including non-stimulants (Atomoxetine, Clonidine, Guanfacine, Viloxazine), Bupropion, or Venlafaxine, as well as respondents whose specific medication class was not reported. Participants exposed to both classes were assigned to the controlled-exposed group to reflect their regulatory exposure profile. A total of 141 adults met the inclusion criteria, with 95 in the controlled-exposed group and 46 in the non-controlled-exposed group.

#### 2.2.2. Outcome

The primary outcome of this study is a binary indicator of whether respondents experienced difficulty in having an ADHD prescription filled due to medication unavailability.

#### 2.2.3. Social Determinants of Health

Nineteen SDOH variables across four domains are considered: (1) demographic factors (age, gender, race/ethnicity, marital status); (2) socioeconomic status (education, income, employment, difficulty paying medical bills in the past 12 months including costs from doctors, dentists, hospitals, therapists, medications, and home care; worry about ability to pay medical bills if sick or injured; and inability to pay mortgage, rent, or housing bills in the past 12 months); (3) neighborhood and built environment (census region, metropolitan status, lack of reliable transportation, and difficulty running errands alone); and (4) health care access and behavior (having a usual place for care, insurance reimbursement for ADHD diagnostic or treatment costs, telehealth use for ADHD prescriptions, difficulty obtaining a prescription due to cost, and use of non-prescribed online medications).

Together with “Controlled exposed”, we have 20 predictor variables. The variable names and their corresponding definitions in the RSS-2 data can be found in Appendix A Table A1. Their count summary is listed in the following table (Table 1) in Section 3.

### 2.3. Statistical Analysis

All invalid responses—such as skipped items or implied refusals, were treated as missing values. Missingness was low, ranging from 0% to 2.8% per variable (0.71% of all predictor values overall), and is reported for each variable in Table 1. For these missing predictor values, we applied a Random Forest–based iterative imputation approach (MissForest-style) to generate data that can be used in machine learning models [26]. It is implemented using scikit-learn’s IterativeImputer with a RandomForestRegressor estimator. The imputed values were then rounded to the nearest integer to preserve the original categorical scales. Chi-square independence tests were conducted to assess differences in the distribution of SDOH and prescription-related variables between the controlled-exposed and non-controlled-exposed groups, with the statistical significance set at *p* < 0.05.

Prior to multivariate modeling, the multicollinearity among predictors was examined using variance inflation factors (VIF) to ensure stable coefficient estimation. Predictors exceeding the conventional threshold of 10 were evaluated individually and retained if they were considered meaningful to the study context [27]. Multivariate logistic regression (MVLR) was then fit as the benchmark model. All 13 participants reporting non-prescribed online medication use reported access difficulty, producing complete separation for this variable; its MVLR coefficient is therefore uninterpretable and is flagged in the regression results (Section 3.4). To capture potential non-linear relationships and identify key predictors of prescription difficulty, five ML algorithms were trained: Random Forest (RF), support vector machine (SVM), elastic net regression, lasso regression (L1), and ridge regression (L2). Lasso, ridge, and elastic net provide regularized linear estimation suited to correlated SDOH predictors, with lasso performing variable selection, ridge stabilizing estimation under collinearity, and elastic net balancing both behaviors. SVM offers strong small-sample generalization and can model non-linear boundaries. RF captures nonlinearities and interaction effects while providing variable importance rankings. The data were partitioned into an 80% training set and a 20% held-out test set. Hyperparameter tuning was performed on the training set using randomized search with five-fold stratified cross-validation, optimizing AUC-ROC across all models. Each pipeline included standard scaling prior to classification, and the best-performing configuration was refitted on the entire training set. Given the small sample size (*N* = 141), overall model performance was subsequently estimated on the full analytic sample using the tuned models, as performance estimates from a single small test set (*n* = 28) are inherently unstable. Mean metrics across the five cross-validation folds are reported in Section 3.5. Permutation importance was computed on the held-out test set using the Random Forest model, which achieved the highest F1 score among all models, to assess each predictor’s contribution to model predictions [28]. All analyses were unweighted. RSS-2 calibration weights are designed to produce nationally representative estimates, whereas this study examines associations within a small analytic subsample (*N* = 141), in which highly variable weights can substantially inflate variance. Moreover, the NCHS quality profile for RSS-2 reports that calibration weighting reduced bias for most benchmark variables but increased it for some, most frequently among healthcare access measures [29]. Estimates should therefore be interpreted as associations within the analytic sample rather than national estimates.

All analyses were performed in Python 3.12 (Python Software Foundation, Wilmington, DE, USA).

## 3. Results

### 3.1. Descriptive Statistics of Studied Population

The patient population sample in this study comprises 141 participants, of whom 95 (67.4%) are in the controlled-exposed group and 46 (32.6%) are in the non-controlled-exposed group. The sample is nearly evenly split by gender (51.8% female, 48.2% male) and is predominantly White (66.0%), with Hispanic (20.6%), Black (6.4%), and other racial and ethnic groups (6.4%) also represented. The majority of participants are between 25 and 44 years of age (62.4%), followed by those aged 18–24 (15.6%), 45–59 (15.6%), and 60–70 (6.4%). Regarding marital status, 40.4% are married, 38.3% have never married, and 18.4% are divorced or separated. Most participants resided in metropolitan areas (85.8%). Indicators of financial strain are prevalent: 44.7% reported difficulty paying medical bills in the past 12 months, 37.6% are unable to meet basic living expenses, and 28.4% are very worried and 42.6% are somewhat worried about affording medical care in the event of illness. Income is broadly distributed across all brackets. With respect to healthcare access, 84.4% reported using telehealth services to obtain ADHD prescriptions, and 93.6% had insurance coverage for ADHD-related costs; however, 36.2% reported difficulty obtaining prescriptions due to cost, 31.9% lacked reliable transportation, and 9.2% reported use of non-prescribed online medications. Full demographic and SDOH characteristics are presented in Table 1.

### 3.2. Association Between SDOH and Medication Access Difficulty

Chi-square tests of independence were conducted between each predictor and medication access difficulty (MEDDIFF). Results are presented in Table 2. Controlled medication exposure is the strongest correlate (*χ*^2^ = 11.230, *df* = 1, *p* < 0.001), indicating that participants using controlled substances are substantially more likely to report prescription access difficulty. Financial barriers also emerge as significant correlates: difficulty paying medical bills in the past 12 months (*χ*^2^ = 8.070, *df* = 1, *p* = 0.004) and worry about paying medical bills if sick (*χ*^2^ = 6.404, *df* = 2, *p* = 0.041) are both associated with greater access difficulty. Age is significantly associated with MEDDIFF (*χ*^2^ = 9.978, *df* = 3, *p* = 0.019), as is non-prescribed online medication use (*χ*^2^ = 5.616, *df* = 1, *p* = 0.018). Lack of reliable transportation approaches but does not reach statistical significance (*χ*^2^ = 3.265, *df* = 1, *p* = 0.071). Remaining variables, including gender, race, income, educational level, metropolitan status, and insurance coverage, showed no significant association with MEDDIFF.

### 3.3. Prescription Access Difficulty by Medication Regulatory Class

As shown in Appendix A Table A3, prescription access difficulty differed markedly by medication regulatory class. Among participants in the controlled-exposed group, 72 of 95 (75.8%) reported difficulty obtaining their ADHD prescription, compared with 21 of 46 (45.7%) in the non-controlled-exposed group, representing a difference of 30.1 percentage points. This difference was statistically significant on a continuity-corrected chi-square test of independence (*χ*^2^ = 11.230, *df* = 1, *p* < 0.001; identical to the Controlled Exposed entry in Table 2), indicating that the regulatory classification of the prescribed medication was strongly associated with downstream prescription fulfillment barriers.

### 3.4. Multivariate Logistic Regression Analysis

Multivariate logistic regression (MVLR) results are presented in Table 3. Three variables reached statistical significance (*p* < 0.05): controlled medication exposure (coefficient = 2.155, *p* < 0.001), difficulty paying medical bills in the past 12 months (coefficient = 1.409, *p* = 0.034), and income (coefficient = 0.145, *p* = 0.042). Participants using controlled medications have substantially higher odds of prescription access difficulty independent of socioeconomic covariates. Remaining variables do not reach significance. Notably, all participants who reported non-prescribed online medication use (*N* = 13 among 141 participants) also reported difficulty obtaining prescriptions.

### 3.5. Model Performance and Feature Importance

**Figure 1:**
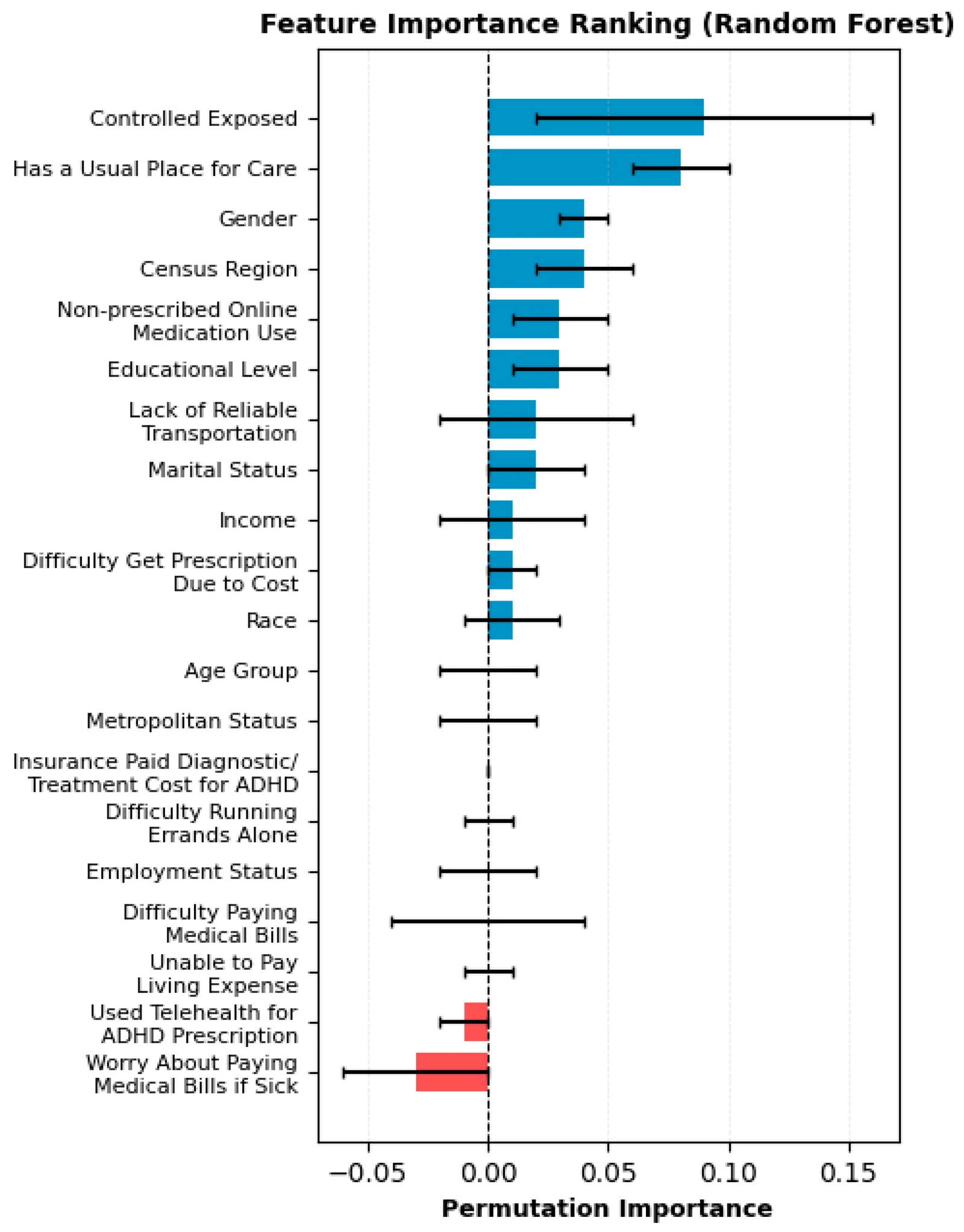
Feature Importance for Random Forest; blue = positive effects, red = negative effects; error bars = standard deviation.

Performance metrics for all machine learning models are presented in Table 4. The Random Forest model achieved the highest recall (0.806) and F1 score (0.772), outperforming the MVLR benchmark (recall = 0.782, F1 = 0.757) across both metrics. SVM and regularized linear models (lasso, ridge, elastic net) showed comparable performance, with F1 scores ranging from 0.732 to 0.742. Ridge regression and Random Forest achieved the highest accuracy (0.688) among all models. Permutation importance rankings derived from the Random Forest model are visualized in Figure 1. Controlled medication exposure and having a usual place for care are the two strongest predictors of prescription access difficulty, followed by gender, census region, and non-prescribed online medication use. Telehealth use for ADHD prescriptions and worry about paying medical bills if sick showed slightly negative permutation importance.

Because permutation importance was computed on a held-out test set of 28 observations, these rankings are subject to sampling variability and should be interpreted as exploratory rather than definitive.

## 4. Discussion

Within a cohort restricted to adults utilizing telehealth for ADHD care, prescription access difficulty was substantially more prevalent among participants using controlled medications than among those on non-controlled regimens (75.8% vs. 45.7%), a difference that persisted in multivariable logistic regression after adjustment for socioeconomic and demographic covariates. This disparity is consistent with a structural gap that aggregate telehealth utilization metrics may obscure: expanding remote access to prescription issuance need not, on its own, resolve the downstream procurement frictions associated with the controlled substance regulatory framework.

These findings are broadly consistent with national cross-sectional estimates, in which 71.5% of stimulant-using adults with ADHD reported fulfillment difficulties attributable to structural unavailability [5]. Claims-based analyses documented that telemedicine flexibilities sustained aggregate stimulant prescribing volumes during the pandemic transition [21], but those data were confined to the point of electronic prescription transmission and could not track whether patients ultimately filled their prescriptions at the pharmacy level. The present analysis extends this literature by linking fulfillment experiences to specific regulatory tiers within a telehealth-using sample, albeit in an exploratory capacity.

The mechanism underlying this disparity is partly traceable to the structural asymmetry between controlled and non-controlled prescribing frameworks. Under 21 CFR § 1306.12, Schedule II controlled substances are categorically prohibited from receiving refills, necessitating a freshly executed prescription for each individual dispensing episode [8]. When manufacturing shortages generate localized pharmacy stockouts, as documented in the 2023 FDA–DEA joint letter describing an estimated market deficit of approximately one billion potential doses in 2022 [9], this prohibition prevents routine inter-pharmacy electronic prescription transfers, forcing patients into a cancel-and-reissue cycle that generates administrative and logistical burdens compounding the search costs of a supply-constrained market. Telehealth’s removal of geographic access barriers does not extend to this downstream fulfillment layer: a remotely issued prescription carries identical regulatory constraints to one issued in person [8,12], leaving the patient to navigate supply shortfalls without the administrative support of a physical clinical encounter.

Multivariable logistic regression identified difficulty paying medical bills in the past 12 months as an independent correlate of prescription access difficulty, echoing prior evidence that adverse social determinants undermine medication adherence [20]. Notably, the same financial strain measure emerged as the strongest SDOH correlate of telehealth utilization itself in a prior analysis of this survey [22], suggesting that financial hardship operates at both the care-access and medication-fulfillment stages of the ADHD treatment pathway. In the context of stimulant shortages, financial and transportation constraints may function as substantive barriers: patients with adequate resources can absorb the temporal and travel costs of searching across multiple pharmacies, whereas those with constrained resources may not [30,31]. Although lack of reliable transportation did not reach statistical significance in the multivariable model (*p* = 0.071), this may reflect limited statistical power.

The positive income coefficient in the multivariable model indicates that higher-income participants reported greater fulfillment difficulty after adjustment for regulatory exposure and financial strain. This counterintuitive direction may reflect greater engagement in active pharmacy searching among resource-rich patients—who persist through, and therefore report, fulfillment friction rather than abandoning treatment [32]—or residual confounding from the bracket-coded income measure treated as a continuous predictor; it should not be interpreted as evidence that affluence impedes access.

Because income is collected by the RSS-2 instrument in 16 ordinal brackets—the native form of the data—and was modeled as a single linear term, potential non-linear threshold effects of financial capability could not be examined; with *N* = 141, disaggregating income into bracket-level indicators would yield small cell sizes and unstable estimates. Formal threshold analyses remain a direction for larger studies.

Non-prescribed online medication use is significantly associated with prescription access difficulty in bivariate testing (*χ*^2^ = 5.616, *p* = 0.018) and carries positive permutation importance ranking of the Random Forest model. All 13 participants reporting non-prescribed online medication use concurrently reported difficulty obtaining their ADHD prescription through formal channels. While this observation derives from a very small subgroup, it is conceptually consistent with regulatory displacement, a process whereby the combined friction of controlled substance prescribing requirements and supply-side shortages redirects patients toward procurement outside regulated supply chains [30]. Such unregulated procurement carries risks related to product authenticity, dosing accuracy, and potential for diversion, including exposure to counterfeit pills containing fentanyl as documented in a 2024 CDC Health Alert [33], representing a distinct public health concern within a population prescribed controlled stimulants.

Having a usual place for care emerged as the second strongest predictor in the Random Forest permutation ranking despite not reaching statistical significance in either the bivariate chi-square test (*p* = 0.098) or the multivariable regression (*p* = 0.268). This divergence suggests that the variable’s contribution may operate through non-linear patterns or interactions with other predictors—structures that tree-based models can exploit but that additive linear specifications cannot. Because the measure distinguishes care settings (physician office, urgent care, or emergency department/other) rather than a simple presence or absence of a usual source, one plausible interpretation is that patients anchored to a physician office receive more consistent prescriber support when navigating stockouts, whereas those relying on episodic care settings do not [34,35]. Given the wide variability of the permutation estimates, this pattern warrants confirmation rather than firm interpretation.

Age is significantly associated with prescription access difficulty in bivariate testing (*χ*^2^ = 9.978, *p* = 0.019), with younger adults disproportionately represented among those reporting fulfillment barriers, possibly reflecting lower financial stability, less established insurance coverage, and greater reliance on telehealth platforms among this cohort [36].

The ML model was conceived as a supplementary descriptive analysis. The Random Forest model achieved the highest recall (0.806) and F1 score (0.772) and reproduced the benchmark ordering by identifying controlled medication exposure as the dominant predictor. The negative permutation importance of worry about paying medical bills likely reflects collinearity with the retained financial strain measures rather than clinical irrelevance, and the negative importance of telehealth use for ADHD prescriptions is consistent with its near-uniform prevalence in this deliberately telehealth-restricted sample (84.4%), which leaves little variance for the model to exploit.

As the federal telemedicine flexibility approaches its scheduled expiration on 31 December 2026 [13], these findings suggest that downstream fulfillment barriers merit consideration alongside upstream access policies in ongoing regulatory deliberations. The absolute refill prohibition for controlled substances under pharmaceutical supply shortage conditions represents one pressure point worth examining; for instance, whether limited inter-pharmacy transfer provisions might reduce administrative burden when a patient’s usual pharmacy cannot fill a prescription. Pharmacy benefit managers and managed care organizations are well-positioned to identify patients at elevated fulfillment risk through formulary management, medication utilization programs, and targeted clinical interventions, and to facilitate access to therapeutically equivalent non-controlled alternatives when stimulant supply is constrained [37,38]. Integrating brief SDOH screening, particularly for financial strain and transportation barriers, into telehealth workflows could additionally help identify at-risk patients before a prescription is issued, though prospective evaluation would be necessary before adoption. These observations generate hypotheses and should be understood as a descriptive basis for regulatory and clinical deliberations.

### Limitations and Future Directions

Several limitations of this study warrant acknowledgment. First, the cross-sectional design precludes causal inference; the observed associations between regulatory classification, SDOH factors, and prescription access difficulty should not be interpreted as causal relationships. Second, the relatively small studied sample (*N* = 141; 95 controlled-exposed, 46 non-controlled-exposed) constrains statistical power, increases the instability of multivariable estimates, and limits the generalizability of machine learning performance metrics. The small sample also precluded disaggregating Schedule II from Schedule IV users, whose refill rules and shortage exposure differ; controlled-exposed estimates therefore predominantly reflect Schedule II stimulant users. Third, unmeasured confounders, including insurance formulary restrictions, local pharmacy chain coverage, and individual prescriber practice patterns, may influence fulfillment experiences in ways not captured by the RSS-2 instrument. Fourth, the low RSS-2 panel response rates introduce the possibility of nonresponse bias. Because analyses were unweighted, these estimates characterize the analytic sample rather than nationally representative values. The studied sample was deliberately restricted to adults with active telehealth utilization to evaluate downstream fulfillment barriers within the specific regulatory context of remote prescribing; findings should accordingly be interpreted as characterizing the telehealth-mediated ADHD care pathway and not generalized to populations receiving exclusively in-person treatment. Fifth, complete separation of the non-prescribed online medication use variable (all 13 such participants reported access difficulty) rendered its multivariable coefficient uninterpretable; this pattern is therefore interpreted descriptively rather than inferentially. Future research should prioritize longitudinal study designs with larger, population-representative samples to enable formal causal inference and to track fulfillment trajectories across regulatory transitions; prospective work incorporating pharmacy-level dispensing records alongside patient-reported outcomes would allow researchers to disentangle supply-side stockout effects from individual-level SDOH barriers with greater precision, and age-stratified analyses would further clarify whether younger adults face systematically distinct fulfillment barriers under the current controlled substance telehealth framework.

## 5. Conclusions

This study suggests that expanding telehealth access does not, by itself, resolve the downstream procurement frictions associated with the controlled substance regulatory framework. Adults using controlled ADHD medications via telehealth faced substantially higher rates of prescription access difficulty than those on non-controlled regimens, with financial strain further compounding this disparity. Multivariable regression identified controlled medication exposure, income, and difficulty paying medical bills as independent correlates of access difficulty, and Random Forest permutation importance likewise ranked controlled medication exposure as the dominant predictor. As federal telemedicine flexibility approaches its 31 December 2026 expiration, these findings point to the need for targeted policy interventions addressing both regulatory and socioeconomic determinants of medication access to ensure equitable continuity of ADHD care.

## Figures and Tables

**Table 1:** Baseline Characteristics of the Analytic Sample (*N* = 141).

Variable	Count, *n* (%)	Missing, *n* (%)
**Gender**		0 (0.0)
Male	68 (48.2)	
Female	73 (51.8)	
**Age Group**		0 (0.0)
18–24 years	22 (15.6)	
25–44 years	88 (62.4)	
45–59 years	22 (15.6)	
60–70 years	9 (6.4)	
**Race**		1 (0.7)
White	93 (66.0)	
Black	9 (6.4)	
Other	9 (6.4)	
Hispanic	29 (20.6)	
**Marital Status**		4 (2.8)
Married	57 (40.4)	
Divorced/Separated	26 (18.4)	
Never married	54 (38.3)	
**Census Region**		0 (0.0)
Northeast	20 (14.2)	
Midwest	32 (22.7)	
South	54 (38.3)	
West	35 (24.8)	
**Unable to Pay Living Expenses**		2 (1.4)
No	86 (61.0)	
Yes	53 (37.6)	
**Difficulty Paying Medical Bills**		0 (0.0)
No	78 (55.3)	
Yes	63 (44.7)	
**Worry About Paying Medical Bills if Sick**		1 (0.7)
Very worried	40 (28.4)	
Somewhat worried	60 (42.6)	
Not at all worried	40 (28.4)	
**Income**		0 (0.0)
Less than $5000	5 (3.5)	
$5000 to $9999	7 (5.0)	
$10,000 to $14,999	2 (1.4)	
$15,000 to $19,999	3 (2.1)	
$20,000 to $24,999	8 (5.7)	
$25,000 to $29,999	3 (2.1)	
$30,000 to $34,999	4 (2.8)	
$35,000 to $39,999	7 (5.0)	
$40,000 to $49,999	12 (8.5)	
$50,000 to $59,999	8 (5.7)	
$60,000 to $74,999	16 (11.3)	
$75,000 to $84,999	15 (10.6)	
$85,000 to $99,999	12 (8.5)	
$100,000 to $124,999	17 (12.1)	
$125,000 to $149,999	9 (6.4)	
$150,000 or more	13 (9.2)	
**Employment Status**		3 (2.1)
Unable to work/Other	54 (38.3)	
Working	84 (59.6)	
**Educational Level**		0 (0.0)
High school or less	39 (27.7)	
Some college	53 (37.6)	
Bachelor’s or higher	49 (34.8)	
**Lack of Reliable Transportation**		2 (1.4)
No	94 (66.7)	
Yes	45 (31.9)	
**Metropolitan Status**		0 (0.0)
Non-metro	20 (14.2)	
Metro	121 (85.8)	
**Has a Usual Place for Care**		0 (0.0)
Doctor’s office/health center	80 (56.7)	
Urgent care	10 (7.1)	
Emergency room/other	51 (36.2)	
**Difficulty Getting a Prescription Due to Cost**		1 (0.7)
No	89 (63.1)	
Yes	51 (36.2)	
**Non-prescribed Online Medication Use**		3 (2.1)
No	125 (88.7)	
Yes	13 (9.2)	
**Difficulty Running Errands Alone**		2 (1.4)
No difficulty	77 (54.6)	
Some difficulty	41 (29.1)	
A lot of difficulty	20 (14.2)	
Cannot do it	1 (0.7)	
**Used Telehealth for ADHD Prescription**		0 (0.0)
No	22 (15.6)	
Yes	119 (84.4)	
**Insurance Paid Diagnostic/Treatment Cost for ADHD**		1 (0.7)
No	8 (5.7)	
Yes	132 (93.6)	
**Controlled Exposed**		0 (0.0)
No (non-controlled only)	46 (32.6)	
Yes (controlled and/or mixed)	95 (67.4)	

Note: Percentages are calculated using the full analytic sample (*N* = 141) as the denominator; category counts may not sum to the total due to missing responses (skipped items or implied refusals). Missing, *n* (%) reports each variable’s missing values prior to imputation.

**Table 2:** Chi-square heterogeneity by medication access difficulty.

Variable	*χ* ^2^	*df*	*p*-Value
Controlled Exposed	11.230	1	<0.001 *
Difficulty Paying Medical Bills	8.070	1	0.004 *
Lack of Reliable Transportation	3.265	1	0.071
Difficulty Getting a Prescription Due to Cost	3.403	1	0.065
Age Group	9.978	3	0.019 *
Worry About Paying Medical Bills if	6.404	2	0.041 *
Sick			
Non-prescribed Online Medication Use	5.616	1	0.018 *
Employment Status	2.985	1	0.084
Has a Usual Place for Care	4.652	2	0.098
Marital Status	4.106	2	0.128
Metropolitan Status	1.383	1	0.240
Insurance Paid Diagnostic/Treatment	0.035	1	0.852
Cost for ADHD			
Unable to Pay Living Expense	1.059	1	0.303
Used Telehealth for ADHD Prescription	0.970	1	0.325
Educational Level	1.195	2	0.550
Census Region	1.688	3	0.640
Race	1.626	3	0.654
Income	11.281	15	0.732
Difficulty Running Errands Alone	0.562	3	0.913
Gender	0.000	1	1.000

* Statistically significant at the 0.05 level.

**Table 3:** Multivariate Logistic Regression Results for Predictors of Prescription Access Difficulty Among Adults With ADHD Using Telehealth.

Variable	Coefficient	95% CI	*p* > |z|
Gender	0.180	(−0.812, 1.172)	0.722
Age Group	−0.476	(−1.133, 0.181)	0.156
Marital Status	0.245	(−0.293, 0.783)	0.373
Unable to Pay Living Expense	−0.035	(−1.400, 1.330)	0.960
Difficulty Paying Medical Bills	1.409	(0.105, 2.712)	0.034 *
Worry About Paying Medical Bills if Sick	0.193	(−0.505, 0.890)	0.588
Income	0.145	(0.005, 0.284)	0.042 *
Employment Status	0.364	(−0.681, 1.409)	0.494
Education Level	0.077	(−0.644, 0.797)	0.835
Lack of Reliable Transportation	1.108	(−0.301, 2.516)	0.123
Metropolitan Status	−0.822	(−2.229, 0.585)	0.252
Has a Usual Place for Care	−0.280	(−0.774, 0.215)	0.268
Difficulty Getting a Prescription Due to Cost	0.207	(−0.986, 1.400)	0.734
Non-prescribed Online Medication Use	23.166	(–, –)	0.999
Difficulty Running Errands Alone	−0.032	(−0.749, 0.685)	0.930
Used Telehealth for ADHD Prescription	0.989	(−0.400, 2.379)	0.163
Insurance Paid Diagnostic/Treatment Cost for ADHD	−0.460	(−2.690, 1.770)	0.686
Race	−0.110	(−0.506, 0.286)	0.586
Census Region	0.147	(−0.313, 0.607)	0.531
Controlled Exposed	2.155	(1.050, 3.260)	<0.001 *

* Statistically significant at *p* < 0.05 level. The coefficient for Non-prescribed Online Medication Use (23.166, *p* = 0.999) is uninterpretable due to complete separation: all 13 participants reporting non-prescribed online medication use reported access difficulty.

**Table 4:** Machine Learning Model Performance.

Algorithm	F1 Score	Recall	Accuracy	ROC-AUC	Brier
Random Forest	0.772	0.806	0.688	0.727	0.203
MVLR (Benchmark)	0.757	0.782	0.674	0.718	0.241
Ridge (L2)	0.742	0.688	0.688	0.738	0.222
Lasso (L1)	0.739	0.699	0.675	0.746	0.206
Elastic Net	0.736	0.699	0.675	0.742	0.198
SVM	0.732	0.709	0.661	0.718	0.193

Note: All metrics are means across 5 stratified cross-validation folds. Models are sorted by F1 score. Brier score ranges from 0 (perfect) to 1 (worst). Hyperparameter values are reported in Appendix A Table A2.

## Data Availability

The data analyzed in this study are publicly available from the National Center for Health Statistics (NCHS) Rapid Surveys System (RSS) Round 2: ADHD, accessible at https://www.cdc.gov/nchs/rss/round2/ADHD.html (accessed on 4 October 2026).

## Abbreviations

The following abbreviations are used in this manuscript:

ADHD	Attention-Deficit/Hyperactivity Disorder
HHS	Department of Health and Human Services
ML	Machine Learning
RSS	Rapid Surveys System
SDOH	Social Determinants of Health
NHIS	National Health Interview Survey

## Appendix A

**Table A1:** Variable Definitions and Survey Questions.

Variable Name	Survey Question	Variable Name in Article
CONTROLLED_EXPOSED	Use of controlled medications (Schedule II stimulants or Schedule IV agents)	Controlled Exposed
PAY_BILL12M	Had problems paying or unable to pay medical bills in past months?	Difficulty Paying Medical Bills
THC_TRANSPOR	Has lack of reliable transportation kept you from medical appointments, work, or daily needs?	Lack of Reliable Transportation
MED_RXDG12MA	Needed prescription medication but unable to afford it in past months?	Difficulty Get Prescription Due to Cost
MAR_MARITAL	Are you now married, living with a partner, or neither?	Marital Status
HRD_ONLPILLS	Have you ever ordered prescription medications online without a prescription?	Non-prescribed Online Medication Use
P_AGEC_R	Age group	Age Group
ADHD_TELERX	Used telehealth to visit doctor/nurse for ADHD prescription since March 2020?	Used Telehealth for ADHD Prescription
PAY_PAYWORRY	If you get sick or have an accident, how worried about ability to pay medical bills?	Worry About Paying Medical Bills if Sick
EMP_EMPLOY	Did you work for pay at a job or business last week?	Employment Status
ACC_HTHUSUAL	Is there a place you usually go when sick or needing healthcare?	Has a Usual Place for Care
ADHD_INS	Did health insurance pay for ADHD diagnostic or treatment costs in past 12 months?	Insurance Paid Diagnostic/Treatment Cost for ADHD
THC_HOUSCOST	Were you unable to pay mortgage, rent, or utility bills in past 12 months?	Unable to Pay Living Expense
P_RACE_R	Race	Race
P_EDUCATION_I_R	Education level	Educational Level
P_GENDER	Gender	Gender
P_REGION	Region	Census Region
SOC_ERRANDS	Do you have difficulty doing errands alone due to physical, mental, or emotional condition?	Difficulty Running Errands Alone
P_METRO_R	Metro	Metropolitan Status
P_INCOME_I_R	Income	Income

**Table A2:** Hyperparameter Values Selected by Randomized Search.

ML Algorithm	Hyperparameter Values
Random Forest	n_estimators = 50; max_depth = 5; min_samples_split = 2; class_weight = balanced
SVM	kernel = linear; C = 0.037; gamma = auto; class_weight = balanced
Elastic Net Regression	l1_ratio = 0.8; C = 0.336; class_weight = balanced
Lasso Regression (L1)	C = 1.194; class_weight = balanced
Ridge Regression (L2)	C = 0.008; class_weight = balanced

**Table A3:** Prescription Access Difficulty by Medication Regulatory Class.

Outcome	Controlled Exposed (*N* = 95)	Non-Controlled Exposed (*N* = 46)
Had difficulty obtaining ADHD prescription, *n* (%)	72 (75.8%)	21 (45.7%)
